# 

**Published:** 1884-10

**Authors:** 


### CONTENTS:

Art. I.—The Origin of Defective Enamel. (Continued.) By W. H. Eames, D. D. S., 241

Art. II—Tin and Gold Combined as a Filling Material. By W. D. Miller, of Berlin 252

Art. III—The Promotion of Osseous Development. By Dr. Geo. Watt, ............ 257

Art. IV.—A History of Dentistry. By George H. Perine, D. D. S., ............ 263

Art. V.—Antiseptics and Disinfectants. By E. J. Lilly, M. D., ............ 259

Art. VI.—Dental Diagnosis. By A. T. Bigelow, D. D. S., ............ 279

American Academy of Dental Science............ 279

Maryland State Dental Association, ............ 279

### MONTHLY SUMMARY.

Micro-Organism, ............ 281

Treatment of Sick Headache, ............ 283

Prevention of Irregularity, ............ 284

A New Method of Removing Nasal Polypi, ............ 285

An Overdose of Chloral During Parturition, ............ 285

Prevention of Caries and Irregularity, ............ 286

Miryachit—A New Disease, ............ 286

Dangerous Adulteration of Iodoform, ............ 287

Why Negroes are Black, ............ 287

Daily Amount of Milk Necessary for an infant, ............ 288

